# Multifunctional Polymer-Based Graphene Foams with Buckled Structure and Negative Poisson’s Ratio

**DOI:** 10.1038/srep32989

**Published:** 2016-09-09

**Authors:** Zhaohe Dai, Chuanxin Weng, Luqi Liu, Yuan Hou, Xuanliang Zhao, Jun Kuang, Jidong Shi, Yueguang Wei, Jun Lou, Zhong Zhang

**Affiliations:** 1 CAS Key Laboratory of Nanosystem and Hierarchical Fabrication, National Center for Nanoscience and Technology, Beijing, 100190, P.R. China; 2 State Key Laboratory of Nonlinear Mechanics, Institute of Mechanics, Chinese Academy of Sciences, Beijing, 100190, P.R. China; 3 University of Chinese Academy of Science, Beijing, 100049, P.R. China; 4 Department of Materials Science and NanoEngineering, Rice University, Houston, Texas 77005, USA

## Abstract

In this study, we report the polymer-based graphene foams through combination of bottom-up assembly and simple triaxially buckled structure design. The resulting polymer-based graphene foams not only effectively transfer the functional properties of graphene, but also exhibit novel negative Poisson’s ratio (NPR) behaviors due to the presence of buckled structure. Our results show that after the introduction of buckled structure, improvement in stretchability, toughness, flexibility, energy absorbing ability, hydrophobicity, conductivity, piezoresistive sensitivity and crack resistance could be achieved simultaneously. The combination of mechanical properties, multifunctional performance and unusual deformation behavior would lead to the use of our polymer-based graphene foams for a variety of novel applications in future such as stretchable capacitors or conductors, sensors and oil/water separators and so on.

Three-dimensional (3D) macroscopic architectures with controlled conformation and orientation become an efficient way to fully realize the excellent physical properties of individual nanomaterials at macroscopic level. A number of methods, such as hydrothermal and chemical reduction, electrochemical synthesis, template assisted assembly, and chemical vapor deposition have recently been developed to fabricate highly porous nanomaterials-based cellular monoliths^1^. Particularly, coating nanomaterials upon 3D polymer foam (also called sponge)-based templates provides a simple, low-cost and scalable mean for creating robust nanomaterial-based macroscopic foams without compromising intrinsic flexibility^2^,^3^,^4^,^5^,^6^,^7^,^8^,^9^,^10^,^11^,^12^,^13^,^14^,^15^,^16^,^17^,^18^,^19^,^20^,^21^,^22^,^23^,^24^,^25^,^26^,^27^,^28^,^29^. Generally, the polymer-based templates used were commercially available foams, *e.g.* polyurethane (PU) foam^2^,^3^,^4^,^5^,^6^,^7^,^8^,^9^,^10^,^11^,^12^,^13^,^14^,^15^,^16^,^17^ and melamine (PM) foams^18^,^19^,^20^,^21^,^22^,^23^,^24^,^25^,^26^,^27^. Through utilizing these templetes, various types of nanomaterials including organic nanomaterials^4^,^11^,^17^,^22^ metal/metal-oxide nanomaterials (*e.g.* silver nanowire, MnO_2_)^2^,^3^,^7^,^9^,^13^,^15^,^19^,^23^,^25^,^27^, and carbon-based nanomaterials (*e.g.* carbon nanotubes (CNTs), graphene)^2^,^5^,^6^,^8^,^10^,^12^,^14^,^16^,^18^,^20^,^21^,^26^,^28^,^29^ have been explored as building blocks to assemble polymer-based multifunctional monoliths. Among them, graphene has been viewed as one of most versatile candidates because of several advantages, including high surface area, excellent electrical conductivity and mechanical properties^30^,^31^. Uses of these advantages give the prospect for considerable technological improvements in many areas of modern engineering, including flexible supercapacitors^29^, microbial fuel cell electrodes^5^,^6^, sensors^21^, elastic conductors^19^, oil/water separators^18^,^22^, current collector^23^, flame retardant^15^,^24^, monolith for hydrogen generation^26^ and biomaterials^14^,^17^,^25^. In general, within this 3D polymer-based template/nanomaterial synergy system, two major factors can determine the multifunctional performance: i) intrinsic properties of nanomaterials, ii) structural geometry of polymer foams^32^. While these referenced works mainly focus on tuning the properties or types of nanomaterials (factor i), to our best knowledge, no reports exist addressing through tuning microstructural geometry of polymer templates (factor ii), which also represents a universal way to tailor the multifunctional performance.

Recently, utilizing the microstructural topology modulation to optimize the functional properties has been developed in flexible and stretchable electrode systems^33^,^34^,^35^,^36^,^37^,^38^. In this case, simple top-down approach was employed to effectively create precise microstructural geometry. Typically, through compressive buckling process, improved multifunctional performance has been demonstrated and further a variety of exciting dynamic behavior resulting from the designed structure was observed^34^,^35^. For example, by harnessing the mechanical instabilities (*e.g.* buckling) of graphene adhered on a biaxially pre-stretched polymer substrate, graphene films can be crumpled with improved hydrophobicity and tunable transparency as well as potential in artificial muscles due to the stretchability^34^. In addition, such buckling design also provided routes to offer conventional, rigid wafer-based technologies with the ability to be stretched, compressed, twisted, bent, and deformed into arbitrary shapes^36^,^37^,^38^.

Inspired by the ideas of introducing buckled structures to enhance both functional performance and flexibility, here we report a novel strategy for the low-cost, scalable fabrication of a new class of 3D multifunctional polymer-based nanomaterial foams based on a triaxially post-buckling microstructure design. The multifunctional foams are first prepared through self-assembly of individual graphene sheets on a 3D commercial polyurethane (PU) skeleton, and then triaxially compressed to introduce microstructural buckles by a simple top-down processing. The resulting polymer-based graphene foams not only effectively transfer the functional properties of graphene, but also exhibit novel negative Poisson’s ratio (NPR) behaviors due to the presence of buckled structure. Our results show that after the introduction of buckled structure, improvement in stretchability, toughness, flexibility, energy absorbing ability, hydrophobicity, conductivity, piezoresistive sensitivity and crack resistance could be achieved simultaneously. The combination of mechanical properties, multifunctional performance and unusual deformation behavior would lead to the use of our polymer-based graphene foams with NPR as stretchable, flexible smart materials in a variety of applications. More importantly, the buckled structure proposed in this work also offers universal, facile way to design a new class of 3D porous template/nanomaterial monolith, which combine ideal properties for potential applications in 3D flexible electronics, stretchable functional materials. Finally, we also applied such post-buckling strategy into fabrication of polymer-based silver nanowires foams, which showed NPR behavior and demonstrated unusual crack resistance as stretchable conductors.

## Results

**Figure 1:**
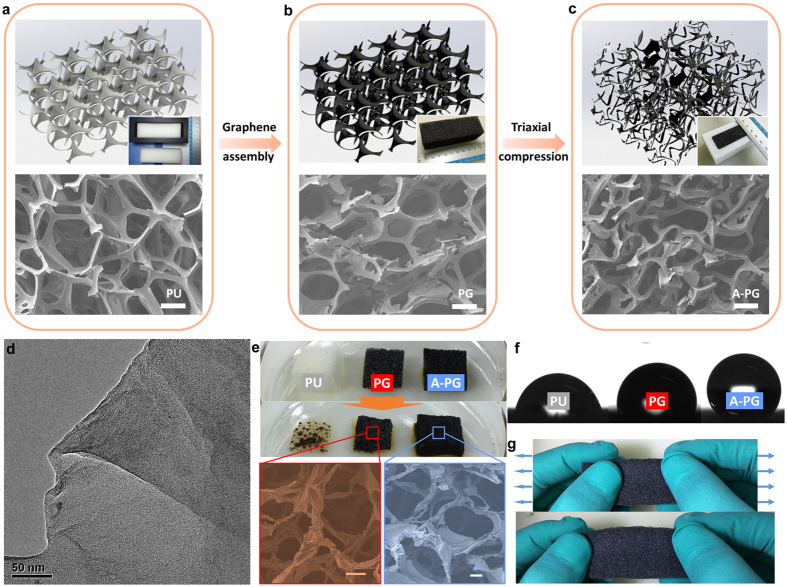
Synthesis and characterization of PG foams and A-PG foams with buckled geometry. (**a,b**) Synthesis of polymer-based graphene (PG, b) foams using polyurethane (PU, a) foams as a three-dimensional scaffold template. (**c**) Mechanically manufacturing process caused triaxially buckled microstructural topology in A-PG foams. From top to bottom: schematic for microstructures, corresponding digital picture of cubic foams and SEM view of their cross-section (scale bars: 300 μm). (**d**) TEM image of graphene stripped from PG foams through dissolving the polymer skeletons. (**e**) Photographs of ignition processes of as-prepared foams in air. Compared to the parent PU and PG foams, A-PG foams hold most of microscopic framework and show much longer burn time without visible smoke during ignition, implying potential application in fire retardancy of graphene coating. Scale bars in the red (PG foam) and blue (A-PG foam) colored SEM view of burned foams is 100 μm. (**f**) Images showing the contact angle of a water drop: 127° on A-PG foams, 112° on PG foams and 84° on parent PU foams. (**g**) Photographs of tension process of A-PG foam indicate its excellent flexibility and negative Poisson’s ratio effect.

The polymer-based graphene foams were prepared by a simple and scalable method, using a combination of chemically self-assembly and mechanically triaxial post-compressing treatment, as illustrated in Fig. 1a–c. Through the “dipping and coating” strategy (Fig. 1b, see *Experimental Section* for details)^2^,^5^,^6^,^7^,^10^,^15^,^18^,^21^, polymer-based graphene (PG) foams were obtained by the assembly of graphene nanosheets onto the commercial PU foams. In efforts to tune the microstructure topology of PG foams, a mechanical compression process in all three directions (triaxial compression) was conducted (Fig. 1c, see *Experimental Section* for details). Note that such triaxial compression treatment in our system is completely different with fracture design reported in Ref. 21, where the foams were compressed with damage in one direction (uniaxial compression) that might be destructive to their mechanical performance (*e.g.* stretchibility). Our triaxially processed foams (now called A-PG foams, where A means auxetic as described later) were compressed (~33%) in all 3 orthogonal directions of parent PG foams cuboid. The cross-sectional view of as-prepared foams is investigated by scanning electron microscopy (SEM). As shown in Fig. 1a, PU foams consist of interconnecting microfibers and the predominant configuration is an array of cells with pentagon with length scale of hundreds of micrometers. During the coating process, the mechanical flexibility of individual nanosheets and their strong affinity for the polymer surface enabled formation of a graphene “skin” that conformed to the surface of PU skeletons^2^,^5^,^6^,^7^,^10^,^15^, The morphology of as-prepared PG foams is similar to that of pure PU foams, but their microfibers change the color from white to black and exhibit a much rough surface due to the presence of graphene sheets “skin” (Fig. 1b and Figure S1a). Accompanying further triaxial post-compression treatment, graphene-wrapped PU microfibers in the as-resulted A-PG foams tend to buckle in order to release the compressive strain (Fig. 1c). Although the microfibers deformed, the graphene nanosheets still tightly adhered to the surface of PU microfibers, which might be related to strong interaction between the graphene sheets and the polymer skeletons^2^,^5^,^6^,^7^,^10^,^15^. The presence of graphene on the polymer microfibers was also characterized by Raman spectroscopy (Figure S1c), which shows the typical G and D bands of reduced graphene oxides (1580 cm^−1^ and 1350 cm^−1^). Close examination near the edge of a cracked microfiber further reveals the layered structures of the graphene “skin” (Figure S1b). The wrinkled, multi-layer sheets could also be easily observed by transmission electron microscopy (TEM) after dissolving the PG foams in dimethyl formamide as shown in Fig. 1d.

As initially mentioned, the bulk physical properties of this 3D PG foam would be affected by the presence of graphene, as well as by the buckling structural geometry of microfibers. The obtained diverse functionality in such multicomponent foams via incorporation of graphene would definitely be tailored by the buckling structure in our systems. Here we present two examples to confirm such effect of buckled structure. First, when the pure PU foam burned in air condition, the pristine polymer frameworks vanish rapidly, whereas the graphene “skin” coated PG foams still remain as an entire framework with apparent structural shrinkage (Fig. 1e). Comparatively, the A-PG foams burned longer and showed less structural shrinkage due to the buckling structure of microfibers in foams. Second, consistent with previous work^8^,^10^,^18^, after assembling graphene sheets on polyurethane skeletons, the obtained PG foams show a contact-angle of 112°, realizing a wettability transformation from hydrophilicity to hydrophobicity, which should be attributed to the intrinsic hydrophobic property of nano-carbon materials. Furthermore, the A-PG foams with buckling structure are found to be more hydrophobic with contact-angle of 127° due to the increase of surface roughness by triaxially reducing the macropore size. Therefore, we suggest that the use of triaxial mechanical compression also represents a facile top-down method to improve hydrophobic performance of 3D polymer-based foams, which are typically prepared only by applying chemical treatment (*e.g.* modulating the chemical properties or types of coating nanomaterials)^3^,^4^,^8^,^10^,^11^,^12^,^17^,^18^,^22^,^24^,^28^,^39^,^40^,^41^,^42^,^43^,^44^,^45^,^46^,^47^.

**Figure 2:**
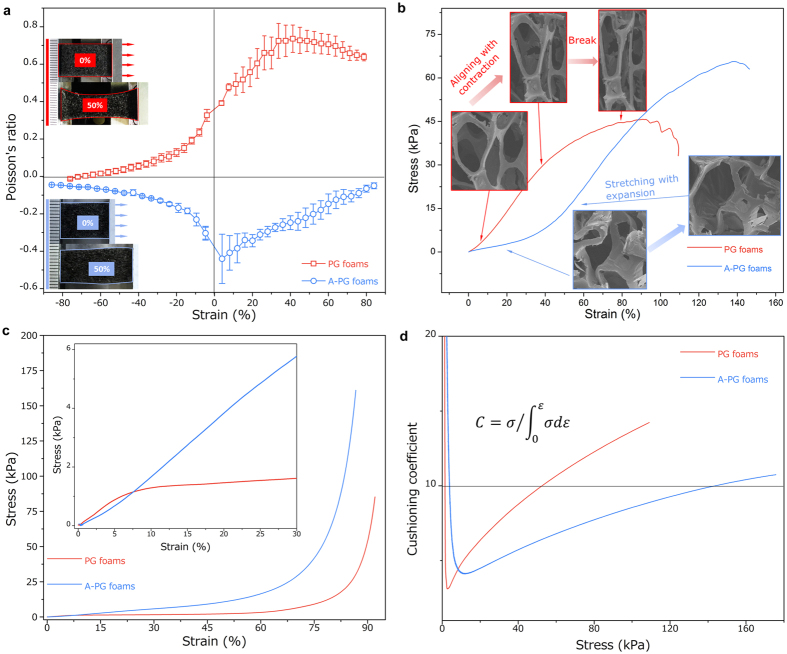
Mechanical characterization of PG foams and A-PG foams. (**a**) Measured Poisson’s ratio versus strain for as-prepared foams (red: PG foams; blue: A-PG foams). Inset: digital picture of PG and A-PG foams under 0 and ~50% strain. (**b**) Measured tensile stress of PG and A-PG foams as a function of strain. Insets show the *in situ* SEM imaging of a representative cell. Compared to PG foams, A-PG foams behave more flexible and stretchable due to rotational deformation of the buckled structure at initial 0–30% tensile strain. (**c**) Measured compressive stress of PG and A-PG foams as a function of strain. When compressive strain is applied up to 30%, Inset shows linear stress–strain curve for A-PG foams whereas two distinct modulus regions are observed for PG foams. All the strain here are engineering strain. two distinct modulus regions are observed. (**d**) Cushioning coefficients of PG and A-PG foams across a stress range of 0–180 kPa. Horizontal dashed line indicates a coefficient of 10.

Despite of multifunctional performance, many of applications such as flexible supercapacitors, pressure sensors and elastic conductors require that the 3D monoliths possess excellent mechanical performance during deformation. And unlike the functionalities mainly stemmed from the coated nanomaterials, the overall mechanical properties of this 3D porous template/nanomaterial system would be dominated by the cellular structures. As shown in Fig. 1g, the most prominent mechanical behaviors that caused by buckling structures within this 3D polymer template/graphene system are the improved flexibility and stretchability under tension, which are not surprising but intriguing for their applications. Moreover, distinctively, the response of the buckled structure in 3D A-PG foams to uniaxial tension also exhibits macroscopically lateral expansion, which is called auxetic behavior (means “expand laterally when stretched”) or negative Poisson’s ratio (NPR) effect. Interestingly, individual graphene nanosheets can be also made auxetic through vacancy defects tailoring^48^, but apparently the negative Poisson’s ratio of A-PG foams should be dominated by the buckling structures of the PU microfibers where the graphene assemblied upon. We thus measured Poisson’s ratios of our samples in Fig. 2a. Similar to that of PU foams in Figure S2, the Poisson’s ratios of PG foams are near +0.3 at small applied tensile or compressive strain level and approach +0.7 in tension and 0 in compression at high strain level. Comparatively, lateral expanding rather than necking is observed for A-PG foams (Fig. 2b insets), showing a minimum Poisson’s ratio of ~−0.5. This unique negative Poisson’s ratio of our A-PG foams could definitely cause the novel lateral expansion behaviors as well as many bulk mechanical properties.

We further explore the influences of buckled structure and its novel NPR behavior on mechanical properties as they are intriguing from an engineering point of view. For example, on the basis of tensile results in Fig. 2b, the A-PG foams represent an almost bilinear behavior, with high flexibility (smaller stiffness) at initial tension, with a stiffness transition between 30% and 50% of strain, meanwhile, with 1.3 times the failure strain, 1.4 times the tensile toughness and 1.5 times the maximum strength of the parent PG foams without buckling structure. Based on *in situ* SEM observation in Fig. 2b insets, we attribute the improvement of both flexibility and strength in A-PG foams to the following facts: i) additional rotational deformation of microfibers at initial tension due to the buckled structures, ii) increased crack resistance caused by buckled structure and NPR effect in A-PG foams (we will discussed later). Under compression (Fig. 2c inset), the PG foams show classical linear-plateau-deification behavior with microfiber-buckling-caused structural collapse starting at ~7% compressive strain. Alternatively, the A-PG foams do not exhibit any plateau region since the microfibers are already buckled and convoluted; instead, they exhibit an extended region (up to more than 50%) of linear elasticity, or resilience. Meanwhile, in Fig. 2c and inset, because of the buckling structures, the improved initial flexibility discussed in tension tests could also be observed under compression as proved by the smaller compressive stiffness for A-PG foams. As a result of this buckled structure induced compression behavior, A-PG foams exhibits improved energy absorption and cushioning performance as shown in Figure S3 and Fig. 2d. We employed a cushioning coefficient (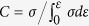) to evaluate the energy absorption property, in which a smaller coefficient means better cushioning behavior^49^,^50^. The stress range with a cushioning coefficient less than 10 has been considerably extended in the coefficient curve of the A-PG foams compared to that of the PG foams, further indicating the effective triaxial compression strategies we utilized for potential mechanical applications such as buffering, damping and energy dissipation structures.

**Figure 3:**
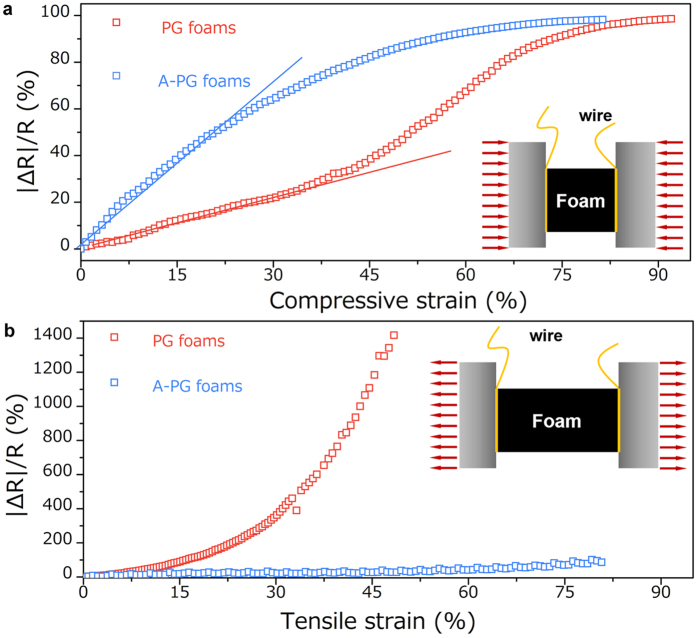
Electrical resistance change of PG foams and A-PG foams under mechanical deformation. (**a**) The resistivity measured on foams was ~100% change at a strain amplitude of 80%, and the initial resistivity-strain relationship is near linear. (**b**) Variation of normalized resistance as a function of tensile strain.

To explore the effects of the buckled structure in our A-PG foams on their functional performance, we further undertook electrical characterizations of our sample in response to mechanical deformation. First, the unique buckled structures of A-PG foams give rise to improved electrical conductivity. As expected, the graphene “skin” that modified onto the polymer skeleton confers conductivity upon the whole surface area of the entire micorfibers network, and the buckled structures further increase the contacts or paths between these conductive network as well as decrease the size of the foams. Indeed, the electrical conductivity of A-PG foams is measured to be 0.001 S/m (Figure S5a), three times higher than that of the parent PG foams. Second, in contrast to the conductive microfiber network in PG foams without post-compression treatment, the buckled microfiber network shows giant variation of the contact area under the compressive deformation, which would determine the piezoresistive sensitivity of conductive foams when utilized in the sensor fields^21^,^51^,^52^. As quantified by the gauge factor 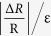 in Fig. 3a, the sensitivity of A-PG foams is calculated to be 2.7, much higher than that of PG foams (0.7). Hence, the post-compression-induced buckling method represents a simple and nondestructive strategy to prepare sensitive cellular solids based flexible sensors (Figure S5b), which were previously prepared by applying destructive fracture on the microfiber in graphene foams^21^. It is worth noting that, A-PG foams also show high electromechanical stability with negligible variation of resistance after 300 cycles (Figure S5d). Third, as aforementioned and shown in Fig. 2b inset, the buckled structures of A-PG foams could offer additional rotational deformation of microfibers at initial tension. It also means that this bucked structures would protect the graphene “skin” from quick disconnection and significant decay in conductivity under stretching. The resistance variations of our samples was studies as a function of tensile strain as shown in Fig. 3b. The resistance increase ΔR/R of A-PG foams with bucked structures at the strain of 80% was 95%, which is much better than that of the PG foams (1600% at 50% strain), PG-foams-PDMS composites (1490% at 60% strain)^19^ and graphene-foams-PDMS composites (ca. 210% at 95% strain, note that structure of this graphen foams structure is quite similar to that of our original PG foams because of the structural similarities in the templates)^53^, and also comparable with that of previously reported melamine (PM)-foam-Ag nanowire-PDMS composites (160% at 100% strain)^19^ and PU-foam-Ag nanowire-graphene-PDMS composites (200% at 50% strain)^13^. Thus, combining with above-mentioned mechanical performance (*e.g.* the improved flexibility and stretchability), the buckled structure we designed in A-PG foams would effectively improve their performance as smart materials.

**Figure 4:**
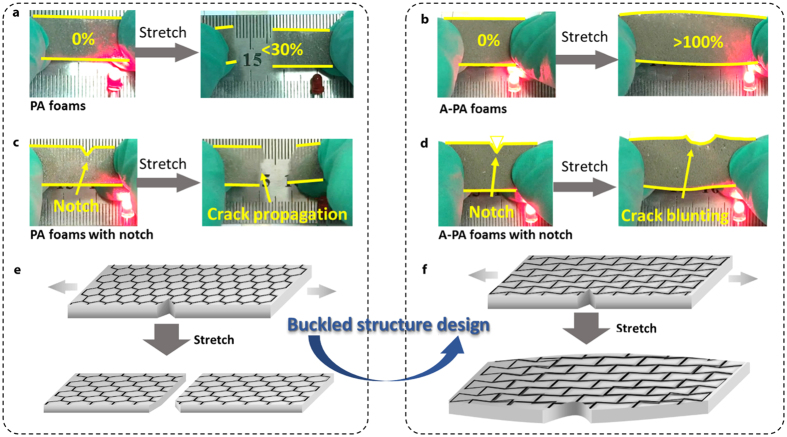
Optical images of the LED lights under the stretching of foams: (**a**) PA foams; (**b**) A-PA foams; (**c**) PA-foams with notch; (**d**) A-PA foams with notch. (**e**,**f**) Schematic illustration of possible crack propagating mechanism of PA foams and crack resisting mechanism of A-PA foams.

We further tried to apply our compression-induced buckled structure into the 3D porous template/nanomaterial monolith beyond the PU-graphene foams. Here, we chose Ag nanowires as our functional nanomaterials because of their high conductivity and their increasingly uses in the field of electronic conductors. Melamine (PM) foams (or sponges) were chosen as our polymer templates as they are another commercially available templates with the desirable physical properties and thus widely used in recent reports^18^,^19^,^20^,^21^,^22^,^23^,^24^,^25^,^26^,^27^. Following quite similar strategy for the fabrication of our PG and A-PG foams, we could easily obtain PM-Ag nanowires (PA) foams by the assembly of Ag nanowires onto the commercial PM foams, and further auxetic PM-Ag nanowires (A-PA) foams with buckled structures by additional triaxial compression processes (see *Experimental Section* for details). Also similar to our testing results of PG and A-PG foams, it is found that as-obtained A-PA foams exhibit prominent improvements in flexibility and stretchability, compared to PA foams. As shown, the PA foams could catastrophically break under less than 30% tensile strain, whereas the A-PA foams with bucked structures showed remarkable tolerance (more than 100%) to tensile deformation. We also integrated LED lights with our samples as simple demonstrations of stretchable conductors in Fig. 4. The brightness of the connected LED showed almost no change after stretching the A-PA samples to more than 100% tensile strain, indicating their high performance as stretchable conductors, comparable with that of PDMS/carbon nanotube network systems in Figure S7a. More interestingly, unlike the PA conductors and the PDMS substrate with positive Poisson’s ratio in Fig. 4c,e and Figure S7b, the auxetic A-PA conductors showed remarkable crack resistance. For example, crack tip in a V-notch of A-PA samples could be effectively shielded as illustrated in Fig. 4f, and crack blunting rather than propagation could be observed in Fig. 4d. Consequently, the buckled structure and NPR deformation behaviors enabled our A-PA conductor more than 100% stretchability even after a shallow V notching, which is intriguing for the stretchable conductors in practice. After discussion of PU-graphene system and this simple demonstration of PM-Ag nanowire system, we, therefore, believe that our 3D triaxial compression method would provide a novel, universal and facile strategy for the fabrication of a new class of high-performance template-based nanomaterials monoliths.

## Discussion

Recently, extensive works have employed the strategy of incorporation various types of nanomaterails in conventional porous templates to fabricate 3D nanomaterials-based macroscopic monoliths for various novel applications. In addition to aforementioned PU and PM foams, the porous templates also includes Ni foams^54^ and inorganic foams^55^ *etc.* Considering the similarities between these reports and our results in structural geometry of templates as well as in self-assembling process, we suggest that besides basic self-assembly methods, the simple top-down post-compression treatment might be combined, by which buckled topographic microstructures and negative Poisson’s ratio effect could be achieved. Through such structural design not only the potential performance of individual nanomaterials might be more effectively realized in macroscopic architecture (at least above-discussed hydrophobicity, conductivity and electrical sensitivity), but also many other bulk mechanical properties can be improved as a result of buckled structures such as toughness, flexibility, stretchablity, damage resistance, which are ideal properties for the use of this next generation of cellular solids in potential applications in 3D flexible electronics, stretchable functional materials and biosystem. Additionally, the bucked structure caused NPR effect further makes the 3D nanomaterial monolith of interests because of the novel behaviors they exhibit under deformation. And we expect that the synergies resulting from functions of nanomaterials and NPR effect will be used widely in future applications such as stretchable capacitors or conductors with high flexibility, shock cushion in body armor, more comfortable (a natural tendency to form dome-shaped surfaces when the Poisson’s ratio became negative) sensor with increased sensitivity and measuring range, easy-to-insert-hard-to-extract stoppers for oil/water separators and so on.

## Methods

### Preparation of Graphene oxide (GO)

GO was prepared from purified natural graphite (obtained from Qingdao Yingshida graphite Co., Ltd., with a particle size of 20 μm) following our previous method^56^. Under agitation, graphite powder (4 g) and sodium nitrate (3 g) were mixed with sulfuric acid (150 mL, 98 wt. %) in an ice bath, and potassium permanganate (18 g) was slowly added to prevent the temperature from exceeding 293 K. The reaction was kept at 293–303 K for 2 h with gas release, and then deionized water (300 mL) was gradually added. The resultant bright-yellow suspension was diluted and further treated with a H_2_O_2_ solution (500 mL, 3%), followed by centrifugation and careful washing to clean out remnant salt. Colloidal dispersions of individual GO nanosheets in water (3 mg/mL) were prepared with the aid of an ultrasonic cleaner.

### Fabrication of PG foams

In a typical process, 100 mL graphene oxide solution (1 mg/mL, water: alcohol = 5:1) was added by 300 μL hydrazine hydrate (80%) and stirred for 2 min at room temperature and then the mixture was poured into a 32 × 32 × 100 mm^3^ nylon vessel. Commercial available PU foams were cut into 30 × 30 × 90 mm^3^ cuboids and cleaned by ethanol and deionized water (DIW) with squeezing manually, followed by drying at 80 °C for 5 hours. The PU foams were immersed into the mixture by squeezing in an ultrasonic cleaner and then the vessel was sealed and placed in an 90 °C oven for the reduction of GO sheets. After 12-hour reduction reaction, a gel-like product was taken out from the vessel. After cleaning by adequate ethanol and DIW and drying at 80 °C to obtain the polymer based-graphene (PG) foams. The density of the resulting foams is about 15 mg/mL with 2 wt% graphene. The PG foams with 2–7 wt% graphene could be obtained through changing the concentration of graphene oxide solution from 1 mg/mL to 3 mg/mL. Samples with 2 wt% graphene were used in most of characterization and only electrical-performance-related characterizations employed the samples with 7 wt% graphene.

### Fabrication of A-PG foams

A Teflon mould with void (inner dimensions 20 × 20 × 60 mm^3^) (Fig. 1a) was employed to introduce buckling structure into PG foam. The PG foam was compressed in all three orthogonal directions with 33% strain after stuffing it into the Teflon mould with the aid of tongue depressor and steel plate. After leaving the compressed foam in the oven for 40 min at softening temperature (180 °C) and cooling within moulds (2 hours) to preserve this new configuration, PG foams with negative Poisson’s ratio (A-PG foams) were prepared.

### Fabrication of PA and A-PA foams

The method for the fabrication of PA and A-PA foams was quite similar to that of PG and A-PG foams. 20 mL silver nanowires (AgNWs) solution (1 mg/mL, water:alcohol = 5:1) was put in an ultrasonic cleaner for 30 min at room temperature and then poured into a 50 × 25 × 25 mm^3^ nylon vessel. Commercial available melamine (PM) foams were cut into 45 × 20 × 20 mm^3^ cuboids and cleaned by ethanol and deionized water (DIW) with squeezing manually, followed by drying at 80 °C for 5 hours. The PM foams were immersed into the AgNWs solution by squeezing in an ultrasonic cleaner and then picked out and transferred into a dry oven at 60 °C oven for 12 hours. After 12-hour, a gray foam was taken out from the oven. Then cleaning by adequate ethanol and DIW and drying at 60 °C to obtain the polymer based-AgNWs (PA) foams. A Teflon mould with void (inner dimensions 13 × 13 × 30 mm^3^) was employed to introduce buckling structure into PM foam. The PM foam was compressed in all three orthogonal directions with 33% strain after stuffing it into the Teflon mould with the aid of tongue depressor and steel plate. After leaving the compressed foam in the oven for 40 min at softening temperature (180 °C) and cooling within moulds (2 hours) to preserve this new configuration, PM foams with negative Poisson’s ratio were prepared (A-PM foams). Then follow the step for preparing PA foams to obtain polymer based-AgNWs (A-PA) foams.

### Structural and electrical characterization of foams

The microstructure and morphology of the as-prepared foams were characterized by SEM (HITACHI S3400). To give an insight of the microstructure, TEM (FEI Tecnai G2 F20 U-TWIN) observations were conducted directly on as-prepared samples. Raman spectroscope (using the 632.8 nm line of a He-Ne laser) was used to examine the presence of graphene on the microfibers of PG foams. For the electromechanical tests, the top and bottom surfaces of the foams were coated with a uniform layer of silver paste and connected by copper wires. During the compression process, the electrical resistance (Keithley 4200 SCS under a bias of 10 mA) was recorded simultaneously.

### Mechanical testing

A dynamic mechanical analyzer (TA, DMA Q800) was used to evaluate the mechanical performance of these foams. The dimensions of all tested samples for compression were about 2 cm × 2 cm × 1 cm and 0.2 cm × 0.5 cm × 2 cm for tension. All the samples were applied an initial load of around 0.01 N in order to provide uniform contact. Static compression/tension tests in Fig. 2 were conducted in the strain ramp mode with a ramp rate of 10% min^−1^. Cyclic strain controlled loading was used to evaluate the dynamic behavior of the PG and A-PG foams with a ramp rate of 200% min^−1^. Dynamic compression testing in Fig. 3c was conducted at 10-μm amplitude, 10–90% strain level, 0.1–100 Hz, 35 °C, a preload of 0.01 N. The Poisson’s ratio caused lateral displacements were measured using a Keyence LKG5001 laser displacement sensor, the vertical distance of recorded point was 18 mm far from the fixed end. The laser was concentrated on the center of a label on the foams to detect the displacement change under deformation.

## Additional Information

**How to cite this article**: Dai, Z. *et al*. Multifunctional Polymer-Based Graphene Foams with Buckled Structure and Negative Poisson's Ratio. *Sci. Rep.* **6**, 32989; doi: 10.1038/srep32989 (2016).

## Supplementary Material

Supplementary Information
