# Supplementary material for: Multifunctional Polymer-Based Graphene Foams with Buckled Structure and Negative Poisson’s Ratio
Source: Sci Rep. 2016 Sep 9;6:32989. doi: 10.1038/srep32989 (PMC5016781; doi:10.1038/srep32989)
Supplement: Supplementary Information [file srep32989-s1.pdf]

SUPPLEMENTARY INFORMATION

Correspondence and requests for materials should be addressed to
Z. Zhang (zhong.zhang@nanoctr.cn); L. Liu (liulq@nanoctr.cn); J. Lou (jlou@rice.edu).

Multifunctional Polymer-Based Graphene Foams with Buckled Structure and Negative Poisson's Ratio

Zhaohe Dai[1,2,4], Chuanxin Weng[1,4], Luqi Liu[1]*, Yuan Hou[1], Xuanliang Zhao[1], Jun Kuang[1,4], Jidong Shi[1,4], Yueguang Wei[2], Jun Lou[3]*, Zhong Zhang[1]*

[1]CAS Key Laboratory of Nanosystem and Hierarchical Fabrication, National Center for Nanoscience and Technology, Beijing, 100190, P.R. China

[2]State Key Laboratory of Nonlinear Mechanics, Institute of Mechanics, Chinese Academy of Sciences, Beijing, 100190, P.R. China

[3]Department of Materials Science and NanoEngineering, Rice University, Houston, Texas 77005, USA.

[4]University of Chinese Academy of Science, Beijing, 100049, P.R. China

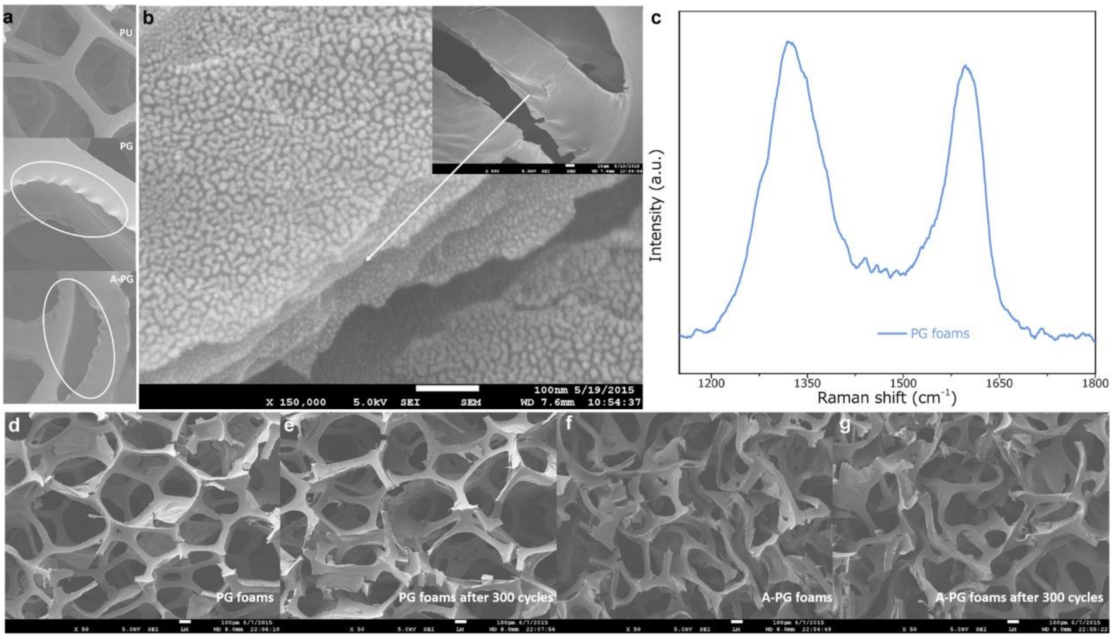

Figure S1. (a) SEM image of PU, PG and A-PG foams (from up tp down). Micrometre-scale crumple could be observed after the coating of graphene in PG and A-PG foams, which might be related to the presence of graphene "skin" and the alcohol caused "expansion-shrinkage" during in situ reduction process. (b) Close examination near the crack tip of a cracked microfiber further reveals the layered structures of graphene sheets assembled around polymer fibers. (c) Characterization on a microfiber of PG foams by Raman spectroscopy shows the typical G and D bands of reduced graphene oxides (1580 cm^{-1} and 1350 cm^{-1}). (d) Typical SEM images of PG and A-PG foams before and after 300 cycles.

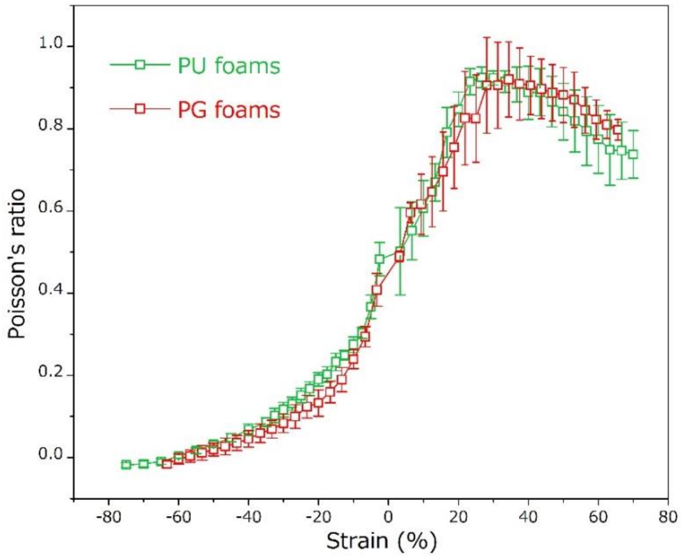

Figure S2. Similar to that of PU foams, the Poisson's ratios of PG foams are near +0.3 for small tensile or compressive strain and approach +0.7 in tension and 0 in

compression at high strain level. The Poisson's ratio of foams is mainly dominated by the skeleton of polymer rather than graphene coating.

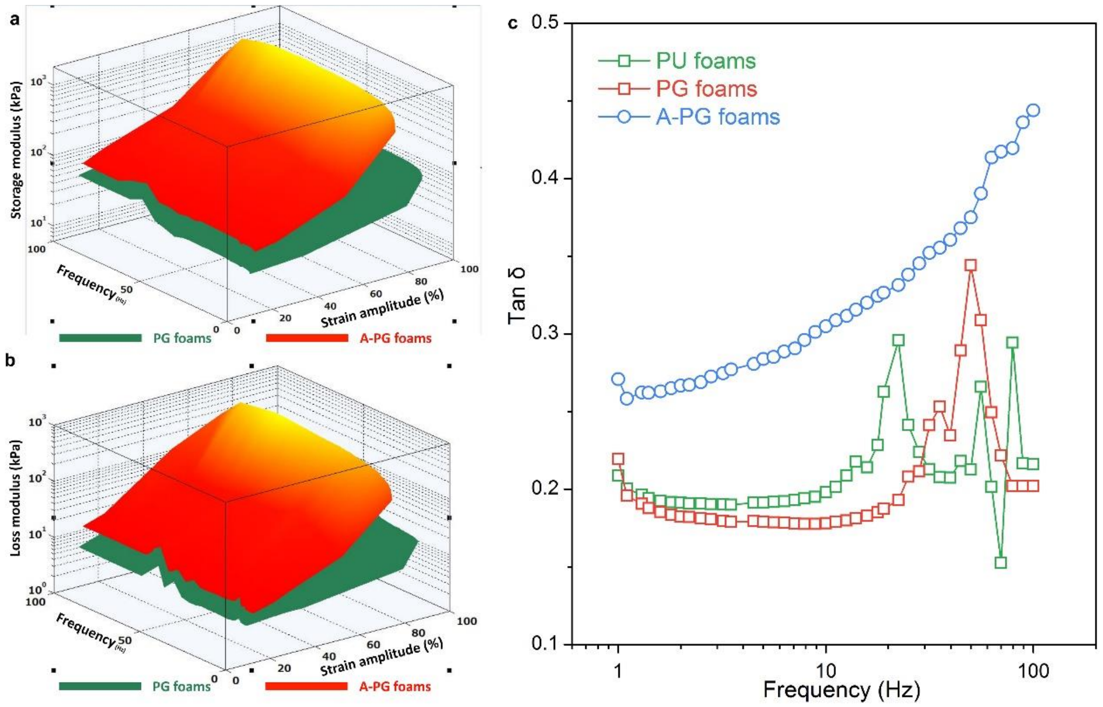

Figure S3. Dynamic compression properties of as-prepared foams. (a) Storage modulus of the PG and A-PG foams mapped as a function of test frequency and strain level. (b) Loss modulus of the PG and A-PG foams mapped as a function of test frequency (0.1-100 Hz) and strain level (10-90%). (c) Loss factor (tan δ) as a function of frequency at high strain level (90%), showing the good energy dissipating capacity of A-PG foams. In terms of the energy dissipation mechanism, the excellent cycling performance (Figure S4) of both PG and A-PG foams rules out the possibility of fracture and friction between broken fragments of microfibers. Instead, the energy loss accompanies with the recoverable behavior of polymeric microfibers as well as highly stacked graphene "skin" during bending/buckling in the compressive loading and the unloading process. During deformation, unlike PU and PG foams, A-PG foams could provide more energy dissipation because of the post-compressing-caused buckling configuration of cells in A-PG foams that maximizes the contact area between neighboring microfibers and generate more frictions.

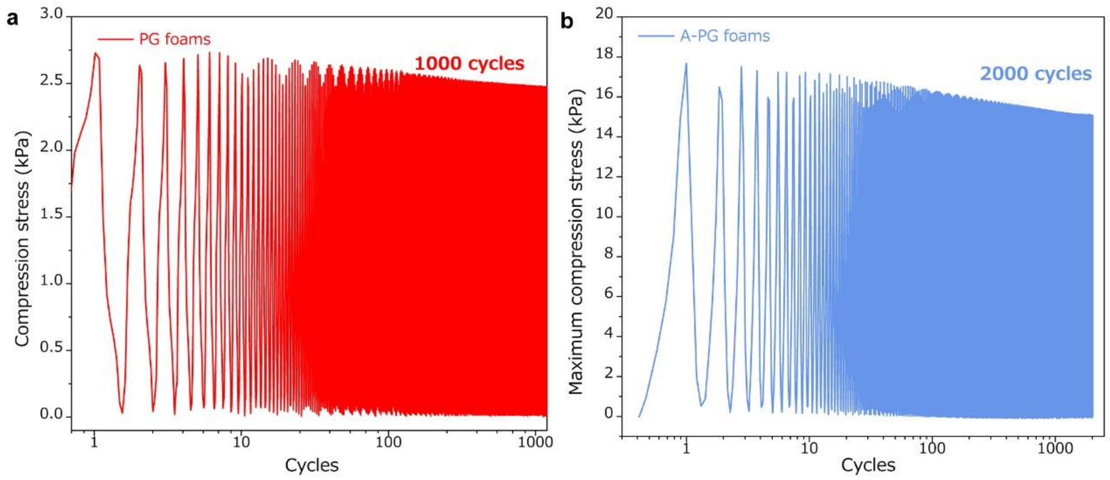

Figure S4. Measured compressive stress response to cyclic compression (0-60% strain) with respect to number of cycles. Compared to PG foams, A-PG foams have higher compressive strength at 60% strain. And both the A-PG foams and PG foams show good mechanical resilience (less than 20% stress relaxation at 60% strain), indicating the stable cyclic performance.

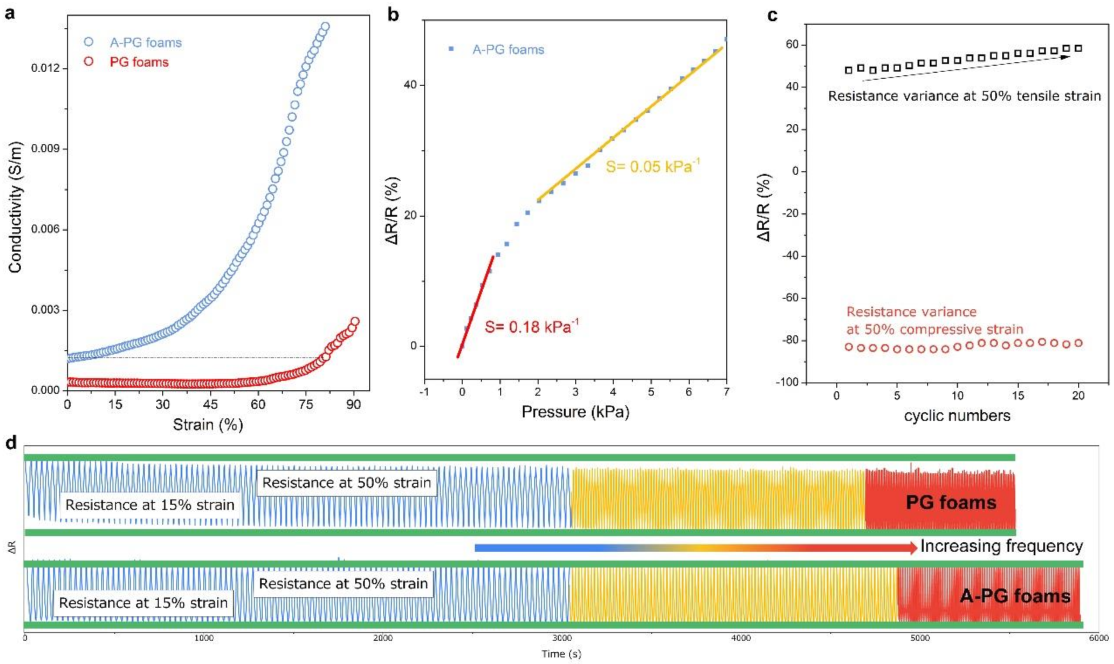

Figure S5. Electro-mechanical coupling behavior of PG foams and A-PG foams. (a) Relative change in conductivity versus strain, derived from the electrical resistance change curves of PG foams and A-PG foams under mechanical compression. (b) The resistance change versus pressure could be derived from the stress-strain curves in Figure 2c and resistance change-strain curves in Figure 3a. The low-pressure region from 0-1 kPa exhibits a slope with a sensitivity of 0.18 kPa^{-1}, while in the large-pressure region (2-7 kPa), the sensitivity of A-PG foam is 0.05 kPa^{-1} (compared to sponges with fracture structures of high pressure sensitivity of 0.23 and 0.03 kPa^{-1} in Ref. 21). (c) The electrical response of A-PG foams corresponding to cyclic numbers at the 50% compressive (negative value as resistance decreases) and tensile (positive value as resistance increases) strain. (d) At the 15-50% strain range, the electrical response corresponding to cyclic loading with increasing frequency. Both the PG and A-PG foams show good stability of electrical properties at 50% strain under hundreds of cycles, while the PG foams show small resistance variation at 15% strain, which might be caused to their less of long-term structural durability such as creep as tested in Figure S6.

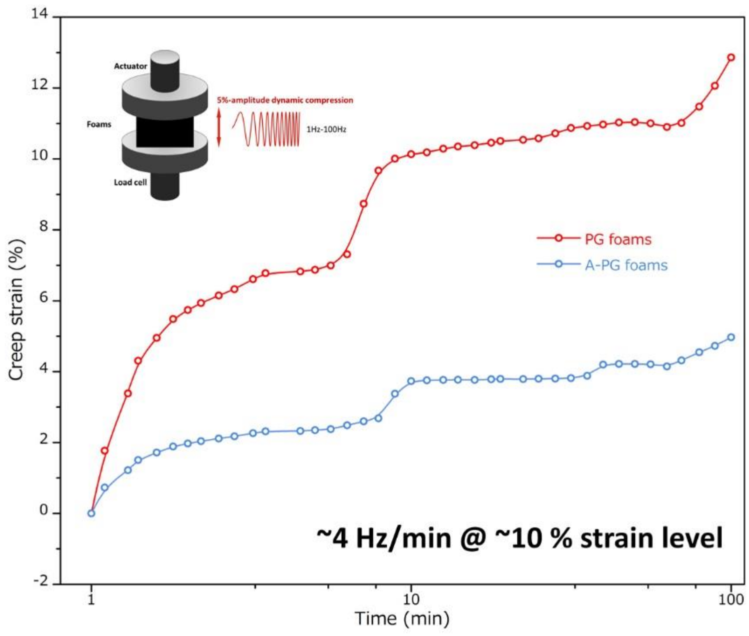

Figure S6. Fatigue strain-time for the PG and A-PG foams. Insert: schematic of compressive cyclic testing. Tests are conducted at room temperature, a strain amplitude of 5%, with a increasing test frequency from 1-100 Hz with a speed of ~4 Hz/min.

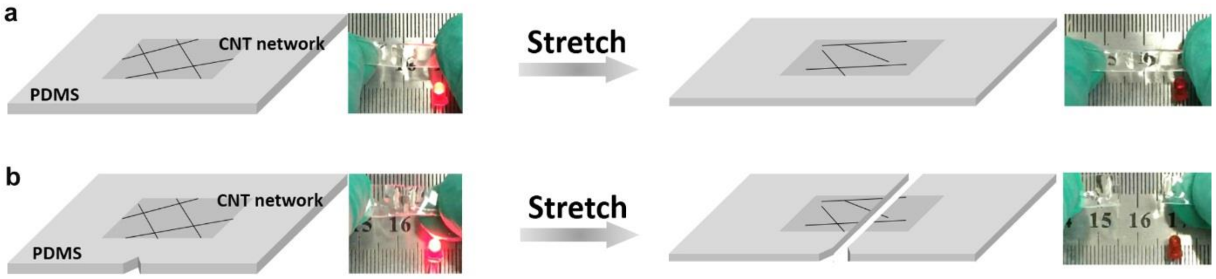

Figure S7. PDMS/CNT network systems as comparisons with samples in Figure 4. Optical images and schematic illustration of the LED lights under the stretching of (a) PDMS/CNT network and (b) PDMS/CNT network with notch in the edge of PDMS substrate.

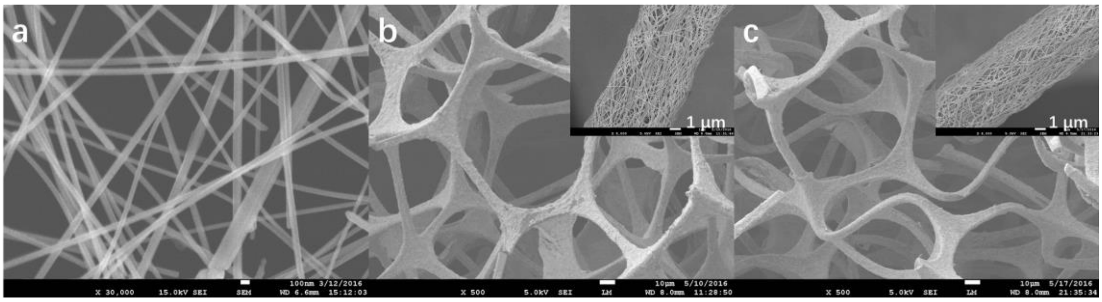

Figure S8. (a) SEM images of as silver nanowires used in our PA and A-PA foams. (b) Typical SEM images of PA foams. Inset: the microfibers with silver nanowires coating. (c) Typical SEM images of A-PA foams. Inset: the microfibers with silver nanowires coating.
